# Moved by Emotions: Affective Concepts Representing Personal Life Events Induce Freely Performed Steps in Line With Combined Sagittal and Lateral Space-Valence Associations

**DOI:** 10.3389/fpsyg.2019.02787

**Published:** 2019-12-11

**Authors:** Susana Ruiz Fernández, Lydia Kastner, Sergio Cervera-Torres, Jennifer Müller, Peter Gerjets

**Affiliations:** ^1^FOM Hochschule für Öekonomie & Management, Essen, Germany; ^2^Leibniz-Institut für Wissensmedien, Tübingen, Germany; ^3^LEAD Research Network, Eberhard Karls University of Tübingen, Tübingen, Germany

**Keywords:** bodily resonance, personal life events, space-valence associations, approach-avoidance behaviors, Body Specificity Hypothesis, free-choice directional step paradigm, generalized estimating equations (GEE), multinomial-Poisson transformation

## Abstract

Embodiment approaches to cognition and emotion have put forth the idea that the way we think and talk about affective events often recruits spatial information that stems, to some extent, from our bodily experiences. For example, metaphorical expressions such as “being someone’s right hand” or “leaving something bad behind” convey affectivity associated with the lateral and sagittal dimensions of space. Action tendencies associated with affect such as the directional fluency of hand movements (dominant right hand-side – positive; non-dominant left hand-side – negative) and approach-avoidance behaviors (forward – positive; backwards – negative) might be mechanisms supporting such associations. Against this background, experimental research has investigated whether positive and negative words are freely allocated into space (e.g., close or far from one’s body) or resonate with congruent (vs. incongruent) predefined manual actions usually performed by joysticks or button presses (e.g., positive – right; negative – left, or vice versa). However, to date, it is unclear how the processing of affective concepts resonate with directional actions of the whole body, the more if such actions are performed freely within a context enabling both, lateral and sagittal movements. Accordingly, 67 right-handed participants were to freely step on an 8-response pad (front, back, right, left, front-right, front-left, back-right, or back-left) after being presented in front of them valence-laden personal life-events submitted before the task (e.g., words or sentences such as “graduation” or “birth of a child”). The most revealing finding of this study indicates that approach-avoidance behaviors and space-valence associations across laterality are interwoven during whole body step actions: Positive events induced steps highly biased to front-right whereas negative events induced steps highly biased to back-left.

## Introduction

The term *bodily resonance* has been increasingly used within embodiment fields in psychology to underline that sensorimotor experiences play a pivotal role in the comprehension of complex phenomena such as emotions (e.g., Fuchs and Koch, 2014). In this regard, it has been suggested that, even at a representational level (e.g., language or thoughts), emotions are tightly bound to their embodied and situated component (e.g., bodily states or action tendencies; Niedenthal, 2007; Barsalou, 2008; Lachmair et al., 2016). Based on this reasoning, a recently discussed topic addresses the idea that the way we think and talk about events with a positive or negative emotional valence often recruits spatial information that stems, to some extent, from our bodily experiences (e.g., Crawford, 2009). For example, “feeling high” or “feeling down” are expressions representing emotional concepts such as “joy” or “sadness” by binding them, in metaphorical terms, with the vertical dimension of space. These metaphorical representations might partly rely on affective bodily states such as upward postures when feeling happy and slumped postures when feeling sad (e.g., Nair et al., 2015), which in turn, may prime associations between emotional valence and verticality (up-positive; down-negative; for an in-depth discussion see Borghi et al., 2017). Experimental findings support these associations by showing that people tend to assign the word “joy” to upper spaces and the word “sadness” to lower ones during tasks that enable freely choosing between different spatial locations (Marmolejo-Ramos et al., 2017). Facilitation effects have also been demonstrated for predefined actions by button-presses “up” or “down” when processing positive or negative words on a monitor as compared to the opposite mappings (up-negative and down-positive; Dudschig et al., 2015; Castaño et al., 2018).

Nonetheless, the way we think and talk in affective terms also suggest space-valence associations beyond verticality. As an illustration, metaphorical expressions such as “being someone’s right hand” or “having two left feet” may link valence-laden concepts such as “relevance” or “clumsiness” with the lateral dimension of space (right-positive; left-negative). Similarly, expressions such as “looking forward to an opportunity” or “leaving bad things behind” may link concepts such as “optimism” or “resignation” with the sagittal space (front-positive; back-negative). Experimental findings, similar to the above-mentioned for the vertical space (mostly based in manual responses) give also support to both of these associations when processing affective concepts (i.e., lateral and sagittal space-valence associations; cf. Milhau et al., 2012). In the following sections, such findings are explained in the light of the Body Specificity Hypothesis (Casasanto, 2009) and approach-avoidance behaviors (e.g., Eder and Hommel, 2013). In real environments, bodily movements are, however, not limited to actions by the hands or arms. Based on this notion, the current study explores whether beyond manual responses, the processing of affective information may also resonate with freely performed whole body movements such as step actions, which are mostly implemented across the sagittal and lateral dimensions. Furthermore, we will examine space-valence associations via whole-body step actions during the processing of information with a personal value for the participants, specifically, personal events with a positive or negative emotional valence, a question underexplored so far.

### Valence Associations Across the Lateral Space: The Affective Role of Hand Dominance

The Body Specificity Hypothesis (Casasanto, 2009, 2014) has become an important reference concerning associations between emotional valence and laterality (right–left). This hypothesis assumes that interactions within the dominant hand-side are more fluent so that they are perceived as more pleasant than interactions within the non-dominant hand-side. Accordingly, right-handers tend to associate the right space with positive valence and the left space with negative valence. In contrast, left-handers tend to show the inverse associations. Children manifest these tendencies too, thus, suggesting that associations between affective valence and right/left spaces form at early stages in life (Casasanto and Henetz, 2012). Based on this reasoning, it has been shown that right-handers describe novel cartoon images as more attractive or happier when they are presented at the right (vs. left) space (e.g., Casasanto, 2009). Moreover, when asked to freely choose between right or left spaces on a sheet of paper, right-handers tend to assign positive stimuli to the right space and negative stimuli to the left space more frequently (e.g., Freddi et al., 2016). Conversely, left-handers show the reversed pattern.

Experimental paradigms have also been developed to investigate space-valence associations of this type when processing affective concepts. Typically, positive or negative words (e.g., “friend” or “war”) are presented in the center of a monitor screen. Participants are required to respond to the stimuli by means of predefined congruent manual actions by button presses on keyboards (e.g., positive – dominant right hand; negative – non-dominant left hand) or incongruent ones (positive – non-dominant left hand; negative – dominant right hand). As a general tendency, responses are more fluent under congruent conditions (e.g., de la Vega et al., 2012, 2013; Kong, 2013). Interestingly, it has been revealed that this sort of effects may also be reflected in button-presses by the right or left foot (de la Vega et al., 2015). Similarly, space-reward associations across laterality have been found when processing reward-related words, showing faster responses under congruent conditions (monetary gain – dominant right hand; monetary loss - non-dominant left hand) than under incongruent ones (monetary gain – non-dominant left hand; monetary loss – dominant right hand; Vicario and Rumiati, 2014). For further spatial-associations across laterality (e.g., luminance, size, letters) see also a review by Macnamara et al. (2018).

Summing up, the processing of affective concepts may resonate with actions by the hands (or feet) in line with space-valence associations postulated by the Body Specificity Hypothesis (e.g., right-positive and left-negative; Casasanto, 2009). Against this background, it is reasonable that space-valence associations across laterality should also be reflected when examining directional actions of the whole body (e.g., to the right or to the left spaces). However, this is an issue that remains very unclear so far. This open issue is even more intriguing when considering that other studies indicate that the processing of emotional stimuli can indeed induce directional actions of the whole body. Yet, these findings are rather related to space-valence associations in line with approach-avoidance behaviors. We address this question in the next paragraphs.

### Valence Associations Across the Sagittal Space: The Affective Role of Approach-Avoidance Behaviors

Approaching positive and avoiding negative stimuli are two of the most general action tendencies in human behavior (e.g., Elliot, 2006). These behaviors are usually linked to the sagittal dimension of space (forward–backwards) due to our visual field unfolding in the front side of the body (cf. Koch et al., 2011). To be more precise, approach behaviors are usually linked to the energy that an agent expends to reduce the distance between positive stimuli and the self. In contrast, avoidance behaviors serve to increase the distance to negative stimuli in order to minimize or prevent their unpleasant effects (cf. Cervera-Torres et al., 2019). Accordingly, it has been suggested that approach-avoidance behaviors may ground affective associations in terms of “close”- positive and “far”- negative (e.g., Van Boven et al., 2010; Davis et al., 2011). There is experimental evidence supporting such associations. For example, akin to findings addressing the lateral (and vertical) space, it has been shown that when asked to freely choose a location within an empty cube positioned in front of them, participants tend to assign positive words (e.g., “open-minded”) at closer areas with regard to their body and negative words (e.g., “insolent”) at farther ones (Marmolejo-Ramos et al., 2018).

Nevertheless, most of the findings supporting space-valence associations in line with approach-avoidance behaviors stem from studies addressing predefined actions rather than freely performed ones. Specifically, participants respond to valence-laden words more fluently (e.g., “intelligent” or “unfriendly”) when they are required to perform congruent actions (positive-approach; negative-avoidance) than incongruent ones (positive-avoidance; negative-approach; for a review see Phaf et al., 2014). To emulate “real” approach-avoidance actions, participants are usually instructed to pull backward or push forward a joystick in response to words presented on a monitor in front of them. It is important to note that the affective meaning of forward-backwards actions is flexibly represented depending on the context of the task itself (e.g., Eder and Hommel, 2013). Backward actions can be perceived as reducing the distance between stimuli and oneself (e.g., one may approach a cup of coffee to the mouth to drink it) whereas forward actions can be perceived as increasing such distance (e.g., repelling an insect from the body; i.e., self-as-reference). Conversely, backward and forward actions can also be perceived as increasing or reducing distance respectively (e.g., withdrawing the hand from an object or guiding the hand forward to grab it; i.e., object-as-reference; Freina et al., 2009).

In the current study, the object-as-reference view is of particular relevance because approach-avoidance behaviors involving whole-body movements seem to follow that contextual representation (for an overview see Bouman and Stins, 2018). For example, some studies use stimuli-response paradigms where valence-laden stimuli are presented on a monitor in front of the participants. Then, they are instructed to step forward or backwards from a central position of a platform placed on the floor, as a function of the presented valence-laden stimuli. In general, facilitation of step actions is favored under congruent conditions (positive stimuli-forward step and negative stimuli-backward step) than under incongruent ones (positive-backward step and negative-forward step; e.g., Stins and Beek, 2011). Nonetheless, rather than affective concepts, the stimuli used in such paradigms are usually more explicit (e.g., emotional pictures). This is an important aspect because, for example, it could be plausible for the word “love” or “unfriendly” to stimulate different mental representations depending on personal experiences (e.g., Niedenthal et al., 2009). Moreover, it has been shown that recalling positive or negative personal life experiences may induce gait patterns in line with approach-avoidance behaviors (e.g., Fawver et al., 2014). Along with this notion, concepts that have personal affective values would be good candidates for examining whether their processing also resonates with movements of the whole body.

Summing up, in the face of the foregoing, one may argue that space-valence research, addressing actions across the lateral and sagittal dimensions of space, has not clarified whether the processing of affective concepts and especially concepts related to personal life events resonate with actions of the whole body. Furthermore, we would also like to draw attention to two important questions that remain unclear:

Firstly, most of the findings described so far, including moving actions by the hands or the whole body, stem from stimuli-response paradigms based on predefined actions. These studies reveal facilitation effects to affective stimuli based on congruent vs. incongruent responses that are framed by the task itself. In other words, the task defines which actions are congruent or incongruent. Hence, participants are not able to decide freely which action to perform. This makes a strong contrast with experimental studies wherein participants are able to assign the affective stimuli to a spatial location freely (e.g., Casasanto, 2009; Marmolejo-Ramos et al., 2017, 2018). Surprisingly, to the best of our knowledge, a free-choice paradigm has not been used to investigate whether the processing of affective stimuli would resonate or induce whole-body movements such as directional steps in line with space-valence associations.

Secondly, space-valence associations in line with the Body Specificity Hypothesis (e.g., right-positive and left-negative for right-handers) and approach-avoidance behaviors (e.g., forward-positive and backward negative) are typically investigated independently from each other. That makes it rather difficult to examine space-valence associations within a more realistic environment wherein bodily movements are not limited to one single direction or even may involve spatial combinations (e.g., front–right, front–left, back–right, or back–left). This lack of research is particularly unfortunate since (a) it could promote a more integrated perspective on space-valence research based on the two afore-mentioned paradigms (paradigms based on predefined step-bodily actions and free-choice paradigms based on stimuli assignments to spatial locations), and (b) it could potentially provide stronger arguments in favor of embodiment views postulating that space-valence associations are implicit and grounded in sensorimotor experiences (cf. Miles et al., 2014). In sum, to date, no free-choice paradigm was used to test space-valence associations in a context where whole body movements may be performed across both, the lateral and sagittal dimensions.

Therefore, the current study investigates space-valence associations by using a novel experimental paradigm based on whole-body movements wherein participants will choose freely in which direction to step across the lateral and sagittal spatial dimensions. It could be expected that a setting where the stimuli are presented in front of the participants might make the saggital dimension more salient than the lateral (cf. Stins and Beek, 2011). Accordingly, we would expect that processing positive stimuli will induce more steps forward whereas processing negative stimuli will induce more steps backward. However, it is also of interest whether the steps are biased to the right or to the left in line with the space-valence associations predicted by the Body Specificity Hypothesis (for right-handers: right-positive and left-negative).

## Materials and Methods

### Participants

A total of 71 participants were recruited through the Online Recruitment System for Economic Experiments (ORSEE; Greiner, 2004) and posts in Facebook groups. Four of these participants were excluded due to their reported left handedness because our main inclusion criteria for the study was right-hand dominance. Therefore, the final sample was composed of 67 right-handed participants aged between 18 and 31 years (*M*_age_ = 24.12, *SD*_age_ = 3.11; 73.1% women). Participants signed an informed consent before the study was conducted. They received course credits or monetary reward for their participation. The experimental testing was in agreement with the guidelines for good scientific practice at the University of Tübingen (Germany) and in accordance with the 1964 Helsinki declaration and its later amendments.

### Stimuli and Apparatus

Once recruited, each participant was asked to report 12 personal emotional life events (e.g., single words or short sentences) via e-mail; six with a positively perceived valence and 6 with a negatively perceived valence. Accordingly, a total of *n* = 804 events were used as experimental stimuli (see Table 1). Participants’ code names were linked together with their reported life events to guarantee their anonymity and to ensure the correct stimuli-participant assignment during the experimental task.

**TABLE 1:** Classification of personal life events into broader categories based on D’Argembeau and Van der Linden (2004).

	**Valence Category**	
	
**Life events**	**Positive (%)**	**Negative (%)**
Leisure activities/parties	24.41	–
Work/Studies success	25.82	–
Romantic relationship	13.62	–
Vacation	11.27	–
Birth of a child	6.10	–
Change of residence	3.29	–
Other	15.49	–
Death/illness of relatives	–	26.29
Failure	–	12.21
End of a relationship	–	10.80
(Own) accident/illness	–	14.55
Quarrel	–	7.04
Unemployment	–	1.88
Other	–	27.23

For the experimental setting it was used a Lenovo ThinkPad T500 Notebook (Intel^®^ Core^TM^ Duo CPU T9600 2 × 2.80 GHz; 4.00 GB RAM; 64 Bit Microsoft^®^ Windows^®^ 7 Professional SP1 operating system) with a monitor [Dell^®^ S2340T 23-inch TFT-LCD monitor; 533.20 mm (height) × 312 mm (width); 1600 × 900 pixel resolution; 0.27 mm pixel distance; 270 cd/m^2^ luminance; 60 Hz refresh rate]. The experimental set-up consisted of the presentation of the positive and negative events in written form (font: Courier New, in white color; size 60) in the center of a monitor with a black background. The monitor was positioned on a table (70 cm height) with a response pad aligned in front of it (Positive Gaming^TM^ Impact Dance Pad; 90 × 80 cm). This pad is a device designed to step toward eight different directions from a central neutral location. Concretely, the pad enabled us to record directional steps from the initial central location toward 4 “absolute” spatial targets (i.e., front, back, right, or left) and 4 “relative” or diagonal spatial targets (i.e., front–right, front–left, back–right, or back–left).

### Procedure

**FIGURE 1:**
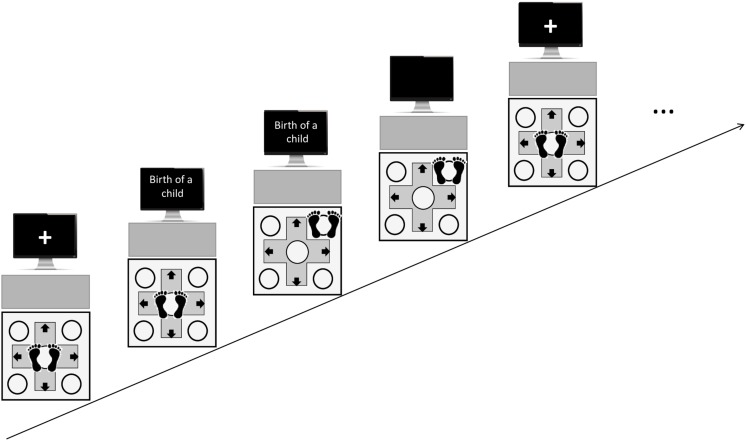
Representation of the experimental procedure. Arrows represent “absolute” spatial targets (front, back, left, and right). Circles represent “relative” or diagonal spatial targets (front–right, front–left, back–right, and back–left).

Participants were kindly asked to take their shoes off and to stand in the middle of the response pad. To become familiar with the experimental procedure, labels representing each spatial target (front, back, right, left, front–right, front–left, back–right, and back–left) were presented twice on the monitor in a randomized order. Participants had to step toward the indicated direction, stand there with both feet and come back to the initial central location on the pad. Each experimental trial started with a fixation cross in the center of the monitor followed by a personal event (e.g., “Birth of a child”). Subsequently, participants were to freely step with both feet toward one of the 8 spatial targets on the pad. Once they stepped on the spatial target, the stimulus disappeared from the monitor and participants returned to the initial location in the center of the pad. A fixation cross reappeared in the middle of the monitor as soon as the participant stood in the middle of the pad (see Figure 1). Then, the next trial was initiated. For each participant, the 12 personal events were presented in randomized order and, for each event, it was recorded one single directional step action.

## Results

**FIGURE 2:**
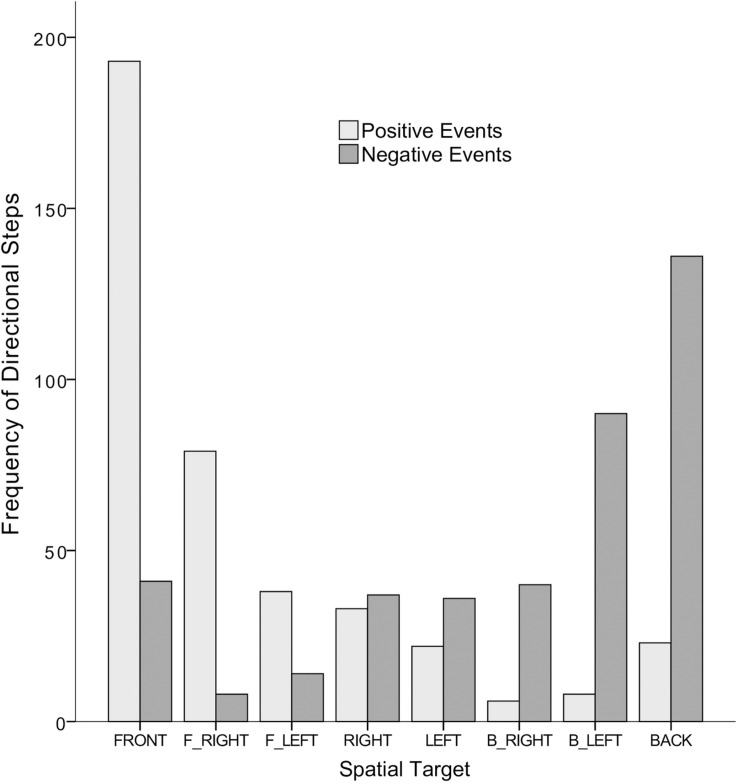
Distribution of directional steps (in raw counts) as a function of the events’ valence category.

The analyses were performed by the Social Package for the Social Sciences (SPSS version 21). Figure 2 shows the distribution of steps associated with valence-laden events.

A chi-square test on the frequency of steps by means of a 2 × 8 crosstabulation between the categorical variables valence category (positive vs. negative) and spatial target (front vs. back vs. right vs. left vs. front-right vs. front-left vs. back-right vs. back-left) revealed a strong significant association between these two factors, χ*^2^*(7,*N* = 804) = 345.41, *p* < *0.001*; Cramer’s *V* = 0.655. *Post hoc* analyses on this crosstabulation indicated that the distribution of steps for positive and negative events within each spatial target was significantly different at the exception of the right and left ones (see Table 2). These effects are in line with the reported adjusted residuals, which indicate the significant contribution of each cell within the whole distribution of steps (values falling above or below 2; *p* < 0.05; see Sharpe, 2015).

**TABLE 2:** Distribution of steps (number and frequencies) together with adjusted residuals and relative odds.

		**Spatial Target**								***Total***
			
		**Front**	**F-right**	**F-left**	**Right**	**Left**	**B-right**	**B-left**	**Back**	
Positive Events	n° Steps	193	79	38	33	22	6	8	23	402
	%_within valence_	48%_a_	19.7%_a_	9.5%_a_	8.2%_a_	5.5%_a_	1.5%_a_	2%_a_	5.7%_a_	
	Adj. Residual	11.8^∗^	8.1^∗^	3.4^∗^	−0.5	−1.9	−5.2^∗^	−8.8^∗^	−10^∗^	
	Rel. Odds	4.71	9.87	2.71	0.89	0.61	0.15	0.089	0.17	
Negative Events	n° Steps	41	8	14	37	36	40	90	136	402
	%_within valence_	10.2%_b_	2%_b_	3.5%_b_	9.2%_a_	9%_a_	10%_b_	22.4%_b_	33.8%_b_	
	Adj. Residual	−11.8^∗^	−8.1^∗^	−3.4^∗^	0.5	1.9	5.2^∗^	8.8^∗^	10^∗^	
	Rel. Odds	0.21	0.10	0.37	0.12	1.64	6.66	11.25	5.91	
*Total*	n° Steps	234	87	52	70	58	46	98	159	804
	%_within valence_	29.1%	10.8%	6.5%	8.7%	7.2%	5.7%	12.2%	19.8%	

*Subscripts of different letters within each spatial target denote significantly different positive (vs. negative) proportion of steps (*p* < 0.05; pair comparisons using *z*-tests with Bonferroni correction). Relative odds = n° steps within positive events divided by n° steps within negative events and vice versa. Values higher than 1 indicate a positive or negative bias (e.g., positive events induced 4.71 times more front steps than negative events did, whereas negative events induced 5.91 times more back steps than positive events did).*

The distribution of steps did not support space-valence associations in terms of positive-right and negative-left, at least in terms of absolute spatial targets (right–left). However, the distribution of steps indicates that processing positive events no only induced a high proportion of front steps (48%) but also front-right steps (19.7%). On the contrary, negative events induced a high proportion of back steps (33.8%) and also back-left steps (22.4%). This results-pattern is congruent with the expected salience of the sagittal dimension, which led to step patterns in line with approach-avoidance behaviors (positive-forward and negative-backward). Yet, at the same time, the results suggest that approach-avoidance behaviors were not the only mechanism driving the results here. The high proportion of front-right (back–left) steps when processing positive (negative) events is in line with a combination of space-valence associations based on approach-avoidance behaviors (positive-front; negative-back) and the body specificity hypothesis (positive-right; negative-left). Furthermore, it is also interesting that the front-right spatial target showed the highest “positive” step bias (steps associated with positive events; 9.87 times more than negative) whereas the back–left spatial target showed the highest “negative” step bias (steps associated with negative events; 11.25 times more than positive). To further explore the strength of these results, we turned to a Poisson regression analysis on the steps counts, which was performed from a generalized estimating equations (GEE) framework^1^ (cf. Guimaraes, 2004). The data modeling included the factors valence category, spatial target, and the interaction between valence category and spatial target. Additionally, participants’ age and gender were used as control covariates. GEE modeling specificities included: (a) a participant’s numerical identifier as a between-subjects variable, (b) trials’ identifier and the factor valence category as within-subject variables with an exchangeable correlation structure, which is appropriate for nested data (cf. Ballinger, 2004), and (c) a robust estimator computation, which accounts for potential misspecifications concerning correlation structures. The results indicate that age [Wald χ*^2^ W*(1) = 0.054, *p* = 0.816] and gender [*W*(1) = 0.032, *p* = 0.857] did not significantly contribute to explain variance related to the directional steps. In contrast, valence category [*W*(1) = 7.28, *p* = 0.007], spatial target [*W*(7) = 70.09, *p* < 0.001], and their interaction *W*(7) = 131.24, *p* < 0.001, were highly significant. Tables 3, 4 show the output related to this interaction.

**TABLE 3:** Poisson regression estimates concerning the distribution of steps.

	***B***	***Standard Error***	***95% C.I. (B)***		***Wald*χ*^2^***	***df***	***Sig.***	***Exp(B)***	***1/Exp(B)***
(Intercept)	–2.124	0.3762	–2.861	–1.386	31.874	1	0.000	0.120	–
Age	–0.001	0.0041	–0.009	0.007	0.054	1	0.816	0.999	–
Gender	–0.006	0.0323	–0.069	0.058	0.032	1	0.857	0.994	–
Valence category	2.290	0.3963	1.513	3.067	33.383	1	0.000	9.875	–
**Spatial target:**									
Front	0.893	0.1666	0.567	1.220	28.746	1	0.000	2.443	–
F-Left	–0.732	0.1755	–1.076	–0.388	17.393	1	0.000	0.481	2.08
Right	–0.873	0.3049	–1.471	–0.275	8.197	1	0.004	0.418	2.39
Left	–1.278	0.2994	–1.865	–0.692	18.234	1	0.000	0.278	3.60
B-Right	–2.578	0.4800	–3.519	–1.637	28.833	1	0.000	0.076	13.16
B-Left	–2.290	0.3923	–3.059	–1.521	34.069	1	0.000	0.101	9.90
Back	–1.234	0.2589	–1.741	–0.727	22.723	1	0.000	0.291	3.44
**Valence × Spatial Target:**									
Valence × Front	–0.741	0.4174	–1.559	0.077	3.151	1	0.076	0.477	2.09
Valence × F-Left	–1.291	0.5329	–2.336	–0.247	5.873	1	0.015	0.275	3.64
Valence × Right	–2.404	0.5226	–3.429	–1.380	21.166	1	0.000	0.090	11.11
Valence × Left	–2.782	0.4648	–3.693	–1.872	35.843	1	0.000	0.062	16.13
Valence × B-Right	–4.187	0.6921	–5.544	–2.831	36.598	1	0.000	0.015	66.66
Valence × B-Left	–4.710	0.6327	–5.950	–3.470	55.429	1	0.000	0.009	111.11
Valence × Back	–4.067	0.5143	–5.075	–3.059	62.530	1	0.000	0.017	58.82

*Estimates of valence category, spatial targets and their interactions are interpreted in relation to the Front-right spatial target and positive events. Negative sign of estimate *B* denotes “less” and positive sign denotes “more.” *C.I.*, Confidence Intervals; *Sig.*, *p*–values.*

**TABLE 4:** Poisson regression estimates concerning the distribution of steps.

	***B***	***Standard Error***	***95% C.I. (B)***		***Wald*χ*^2^***	***df***	***Sig.***	***Exp(B)***	***1/Exp(B)***
(Intercept)	–2.124	0.3785	–2.866	–1.382	31.476	1	0.000	0.120	–
Age	–0.001	0.0041	–0.009	0.007	0.054	1	0.816	0.999	–
Gender	–0.006	0.0323	–0.069	0.058	0.032	1	0.857	0.994	–
Valence category	2.420	0.4018	1.633	3.208	36.288	1	0.000	11.250	–
**Spatial target:**									
Front	–0.786	0.2308	–1.239	–0.334	11.606	1	0.001	0.456	2.19
F-Right	–2.420	0.4052	–3.215	–1.626	35.674	1	0.000	0.089	11.24
F-Left	–1.861	0.3187	–2.485	–1.236	34.093	1	0.000	0.156	6.41
Right	–0.889	0.2374	–1.354	–0.424	14.022	1	0.000	0.411	2.43
Left	–0.916	0.2242	–1.356	–0.477	16.709	1	0.000	0.400	2.5
B-Right	–0.811	0.1800	–1.164	–0.458	20.292	1	0.000	0.444	2.25
Back	0.413	0.1836	0.053	0.773	5.056	1	0.025	1.511	–
**Valence × Spatial Target:**									
Valence × Front	–3.969	0.4740	–4.899	–3.040	70.121	1	0.000	0.019	52.63
Valence × F-Right	–4.710	0.6327	–5.950	–3.470	55.429	1	0.000	0.009	111.11
Valence × F-Left	–3.419	0.5987	–4.592	–2.245	32.607	1	0.000	0.033	30.3
Valence × Right	–2.306	0.5569	–3.398	–1.214	17.144	1	0.000	0.100	10
Valence × Left	–1.928	0.4703	–2.850	–1.006	16.804	1	0.000	0.145	6.89
Valence × B-Right	–0.523	0.4885	–1.481	0.434	1.147	1	0.284	0.593	1.68
Valence × Back	–0.643	0.4595	–1.544	0.257	1.959	1	0.162	0.526	1.90

*Estimates of valence category, spatial targets and their interactions are interpreted in relation to the Back–left spatial target and negative events. Negative sign of estimate *B* denotes “less” and positive sign denotes “more.” *C.I.*, Confidence Intervals; *Sig.*, *p*–values.*

In Tables 3, 4, *Exp(B)* is the natural exponential function of *B* (*e*^B^) and facilitates the interpretation of the results in terms of proportions or likelihoods. In this case, *Exp(B)* related to the factor valence category denotes the relative odds or “positive” (“negative”) step bias associated with the front-right (back-left) targets, i.e., positive events inducing 9.875 times more front-right steps than negative did; negative events inducing 11.25 times more back-left steps than positive did. In the predictor spatial target, *Exp(B)* values inferior to 1 denote *less* proportion of steps when comparing the spatial targets against front-right or back-left. Values superior to 1 denote the opposite. Nonetheless, we also report the inverse function 1/Exp(*B*) reflecting how much the proportion of “positive” (“negative”) steps within front-right (back-left) deviate from the other spatial targets. For example, positive events significantly induced 2.08 times *more* front-right steps than front–left steps. However, positive events also significantly induced 2.44 times *more* front steps than front–right steps. In a similar vein, negative events induced 2.25 times *more* back–left steps than back–right steps. Yet, negative events also induced 1.51 times *more* back steps than back–left steps. Finally, in the interaction term, *Exp(B)* values are interpreted as the odds ratio (OR). For example, the “positive” front–right step bias (9.875) tended to be 2.09 times *higher* than the “positive” front step bias (4.71; marginal effect), which may be explained by the lower number of front-right steps associated with negative events. From the other side, the “negative” back–left step bias (11.25) was significantly superior to the negative bias of the other spatial targets at the exception of back and back–right.

Summing up, the results confirm that: (a) comparing steps’ frequencies associated with positive events only (i.e., light gray columns in Figure 2) the front-right target significantly arose as the second spatial target with higher proportion of steps behind the Front spatial target. In a similar vein, (b) comparing steps’ frequencies associated with negative events only (i.e., dark gray columns in Figure 2) the back-left target significantly arose as the second spatial target with higher proportion of steps behind the Back spatial target. Finally, (c) front–right significantly reflected the highest “positive” step bias, being this effect marginal when compared to the Front target. Interestingly, although descriptively speaking the back-left target also reflected the highest “negative” step bias, this bias was not strong enough to be significantly superior to the negative bias within the back–right and back targets. In any case, it is quite remarkable that front-right and back–left showed the highest OR, namely, the “positive” front–right step bias was 111.11 times higher than the “positive” back–left step bias. The opposite also holds true so that the “negative” back–left step bias was 111.11 times higher than the “negative” front–right step bias.

### Sensitivity Analyses

**FIGURE 3:**
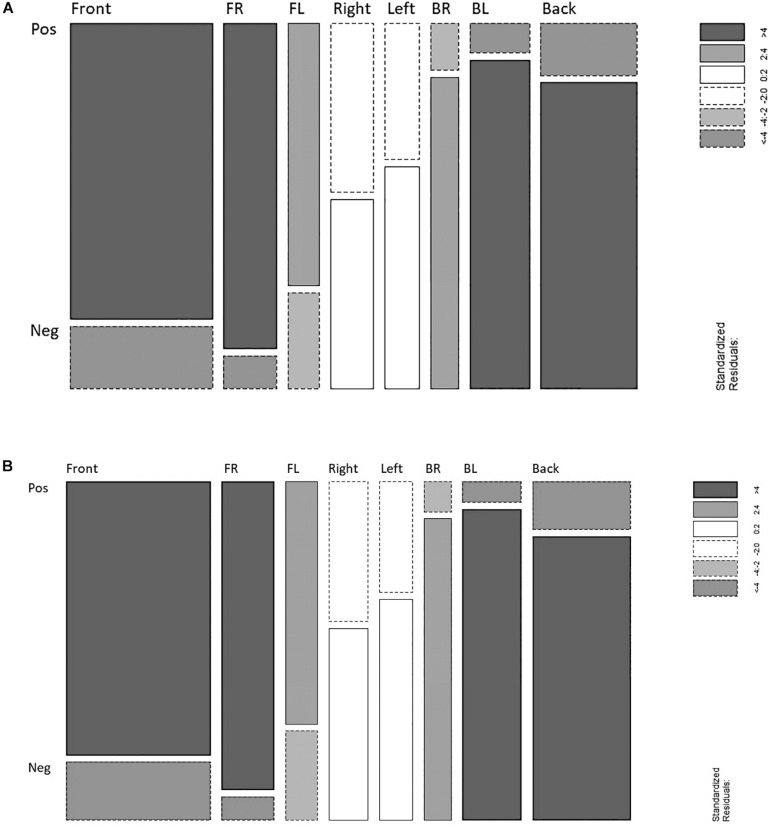
**(A)** Mosaic plot showing distribution of residuals before the exclusion of extreme observations. **(B)** Mosaic plot showing distribution of residuals after the exclusion of extreme observations (for further details on mosaic plots see Hartigan and Kleiner, 1984; Friendly, 1994).

One last important aspect to consider in any regression analysis refers to the inspection of extreme observations potentially affecting the results. Model diagnostics based on Pearson residuals (e.g., Oh et al., 2008) revealed a set of extreme observations representing a total of 16 steps out of 804 (1.9%; within positive events: 6 right, 4 left, 2 back-left, and 2 back-right. Within negative events: 2 front-right; see Figures 3A,B). Using the same Poisson regression model, as above-described, after the exclusion of such observations showed that both, the “positive” front–right step bias and the “negative” back–left steps bias, increased (9.87–12.96 and 11.25–14.89, respectively). Consequently, the “positive” front–right step bias turned to be significantly higher than the “positive” front bias OR = 2.75, *p* = 0.022. Moreover, the “negative” back–left step bias also turned to be significantly higher than the “negative” back step bias, OR = 2.51, *p* = 0.043 but still non-significantly higher than the “negative” back-right steps bias, which is not surprising since both spatial targets were equally affected by the exclusion of extreme observations (Supplementary Tables S1, S2 in Supplementary Material report the complete output of these analyses). It is important to note, however, that the “positive” and “negative” step biases reported in this study are directly related to the number of steps and therefore further studies should investigate whether similar effects can be found, for example, when investigating steps associated with the diagonal dimensions only (front–right, front–left, back–right, and back–left) and/or when increasing the number of affective events. In any case, the current study highlights that front–right and back–left arise as two directions playing an important role during the processing of positive and negative personal life events.

## Discussion

Assuming associations between emotional valence and laterality (for right-handers: right-positive and left-negative; Body Specificity Hypothesis; Casasanto, 2009) and the sagittal dimension of space (close-positive and far-negative; approach-avoidance hypothesis; e.g., Davis et al., 2011) the current study examined whether processing valence-laden concepts, and particularly concepts related to personal life events, could induce directional step actions in line with such associations. Specifically, a novel free-choice directional-step paradigm was used in the present study. This paradigm was inspired by experimental findings indicating that: (a) people tend to freely assign positive and negative concepts such as “happiness” or “unfriendly” to the lateral or sagittal space in line with the mentioned space-valence associations (e.g., Freddi et al., 2016; Marmolejo-Ramos et al., 2018), (b) congruent manual responses have also been shown facilitated after processing affective concepts (rightward-positive and leftward-negative; Milhau et al., 2015 or forward-positive and backward-negative; Freina et al., 2009), and (c) emotional visual stimuli (e.g., pictures) presented in front of participants facilitate congruent approach-avoidance step actions (e.g., forward step-positive; backward step-negative; e.g., Stins and Beek, 2011). However, these findings are based on studies that typically address the lateral and sagittal spatial dimensions independently from each other. Moreover, because in real environments whole bodily actions are not limited to one single movement direction and in some cases could even result in spatial ambiguity (e.g., combinations of front-right, front-left, etc.) the present study investigated whether the processing of valence-laden personal events would resonate with step actions in a setting enabling to freely step toward eight different spatial targets (front vs. back vs. right vs. left vs. front-right vs. front-left vs. back-right vs. back-left). Based on this reasoning, it was hypothesized that positive and negative events presented in front of the participants, would induce more forward and backward steps respectively (approach-avoidance hypothesis). Yet, it was also of interest to explore whether the steps could be biased to the right or to the left in line with the space-valence associations predicted by the Body Specificity Hypothesis (right-positive and left-negative; Casasanto, 2009).

Results show that: (a) positive events induced significantly more front steps whereas negative ones induced more back steps. However, (b) participants did not significantly perform more steps to the right (to the left) after the presentation of positive (negative) life events. Interestingly, the most revealing finding of this study refers to the idea that (c) the sagittal and lateral spatial dimensions were highly associated, resulting in a diagonal spatial dimension with a high front–right step bias after processing positive events and a high back–left step bias after processing negative events. These results are discussed in the following sections.

### Emotional Events Induce Directional Steps in Line With Approach-Avoidance Behaviors

In the present study, positive life events induced step actions more frequently to the front whereas negative life events did so more frequently to the back. These results can be explained in the light of approach-avoidance motivational systems. Approach and avoidance systems facilitate actions that reduce or increase the distance with regard to positive or negative stimuli respectively (i.e., distance regulation mechanism; Seibt et al., 2008). Moreover, approach-avoidance behaviors can also be defined in terms of the reference point within a specific task (e.g., Freina et al., 2009). To be more precise, in the present study, participants were presented personal life events on a monitor in front of them. Subsequently, they had to perform a directional whole-body movement (i.e., step action). As whole-body movements may require external targets (reference points), which are approached or avoided, it is plausible that participants used the computer screen as the reference point. It can be argued then, that this setting elicits a certain distance regulation with regard to the life events that were presented in front of the participants. Accordingly, participants regulated the distance to the emotional events “directly” by performing free approach-avoidance movements in the sagittal spatial dimension. In line with this notion, positive life events led more frequently to forward movements in order to reduce the distance to these events (approach motivation toward the reference) whereas negative life events led more frequently to backward movements in order to increase the distance to these events (avoidance motivation).

These findings are also in line with studies examining gait initiation after the presentation of positive and negative stimuli (e.g., Bouman and Stins, 2018). These studies show, for example, a faster forward gait initiation when participants recalled autobiographical emotional memories related to happiness compared to emotional memories related to sadness (e.g., Fawver et al., 2014), faster step velocity when walked toward pleasant pictures with positive emotional content compared to unpleasant pictures with negative emotional content (Naugle et al., 2010) and a slower forward step initiation when angry faces are presented in front of them than when smiling faces were presented (Stins and Beek, 2011).

### Processing Emotional Events Do Not Clearly Support the Right-Positive and Left-Negative Associations

Although we assumed a stronger activation of the sagittal over the lateral spatial dimension we also expected that, in line with the Body Specificity Hypothesis by Casasanto (2009), participants (all right-handers) will perform more steps toward the absolute right or left spatial targets after the presentation of positive or negative life events, respectively. However, the results of the current study do not support this expectation. Specifically, directional steps toward the right or left spatial targets did not show significant differences after the processing of positive (vs. negative) events. On the contrary, the results suggest that processing the affective events lead, in general, to higher activation of the sagittal space in line with approach-avoidance behaviors.

It is plausible that personal life events, such as the own wedding or the birth of own children, could have been perceived as very vivid or intense (at least more vivid or intense than the commonly used emotional stimuli e.g., emotional words or pictures), thus, stimulating the distance regulation with regard to the stimuli; an effect that could not be achieved when moving to right or left spatial targets. Furthermore, as described in the previous section, the stimuli were presented on a monitor in front of the participants. Therefore, the sagittal space was probably more salient than the lateral space, independent of the influence of the affectivity of the presented life events on step action. In other words, participants could simply have been moving more naturally toward or away from the reference object. However, it is pivotal to highlight that a novelty was introduced in the current study: The experimental setting of the current study did not only consider absolute spatial targets as most space-valence research does (i.e., front, back, right, left) but also considered the combination of those targets that results in two diagonal spatial dimensions (front–right to back–left and front–left to back–right). Although no specific hypothesis was formulated regarding these diagonal spatial dimensions, the result-pattern suggests that they played an important role when participants had to decide in which direction to move after processing the stimuli. These results will be discussed in the next section.

### The Affective Role of the Diagonal Spatial Dimensions

Beyond absolute spatial targets (i.e., front, back, right, and left), participants in the present study were also able to freely step toward four different diagonal spatial targets (front–right, front–left, back–right, and back–left). As described in Table 2, the results reflect that, taking into account only such diagonal targets, positive and negative events induced more steps toward the front-right and back-left directions respectively. This finding suggests that approach-avoidance behaviors (i.e., forward and backward steps) were biased to the right or to the left depending on the emotional valence of the presented event. However, could any mechanism account for such result-pattern?

We argue that these findings are in line with what has been termed as the sword and shield hypothesis (SSH; Brookshire and Casasanto, 2012). This hypothesis assumes behavioral tendencies whereby it is more likely to approach positive stimuli with the dominant hand and to avoid negative stimuli with the non-dominant hand. In fact, the SSH postulates that the wide assumed motivational asymmetries in terms of associations between the brain left hemisphere and the approach system or the right hemisphere and the avoidance system, are indeed derived from studies that mostly rely on the examination of right-handed participants (e.g., Harmon-Jones et al., 2010). Based on this reasoning, the SSH suggests that handedness may be an important factor associated with such motivational asymmetries (e.g., Cretenet and Dru, 2008; Casasanto, 2014). In support of this idea, recent neuroimaging findings indicate that, for right-handers, “approach” is more intensively associated with the left hemisphere and “avoidance” with the right hemisphere, reversing this pattern in left-handed participants (Brookshire and Casasanto, 2012; Brookshire et al., 2013). It is important to note that the use of the right and left hands are contralaterally associated (right hand – left hemisphere; left hand – right hemisphere). It is reasonable, then, to consider the SSH as an extension of the Body Specificity hypothesis wherein right-handers tend to associate the right space with a positive valence, not only because of the fluency of the right hand within the right space but also because of the tendency to approach positive stimuli with the dominant right hand. In contrast, the left space would be associated with a negative valence due to the lesser fluency of the non-dominant left hand and also the tendency to avoid stimuli with this hand. Although to the best of our knowledge, there is no specific evidence supporting these assumptions, the results of the current study could be interpreted in that direction: Positive stimuli induced more front-right steps as a result of the combination of the “positive” approach mechanism and the “positive” right space association. Conversely, negative stimuli induced more back–left steps as a result of the combination of the “negative” avoidance mechanism and the “negative” left space association.

### Limitations and Future Research Directions

We acknowledge that, as in any piece of research, the findings in the current study face some limitations:

Firstly, all participants in the current study were right-handed. This restriction does not allow to generalize our findings to the whole range of space-valence associations proposed by the Body Specificity Hypothesis (Casasanto, 2009). Concretely, we would not expect differences between left- and right-handers regarding the absolute forward or backward steps associated with approach-avoidance behavior. The same would hold for the absolute lateral spatial targets (right–left) since the sagittal spatial dimension was more salient in our study. However, it could be hypothesized that, as discussed in the section above, left-handed participants would perform more steps toward the front–left direction after processing positive events and toward the back–right direction after processing negative events. Accordingly, it would be interesting to explore whether our findings concerning the diagonal spatial dimension would be replicated for left-handed participants.

Secondly, in line with the just mentioned first limitation, the experimental setting in our study probably caused participants to perceive the monitor as a reference point. Therefore, it is to assume that the setting stimulated mechanisms of distance regulation with regard to the presented emotional life events (i.e., approaching or avoiding the stimuli presented in front of them) rather than lateral movements. Accordingly, it would be necessary to investigate whole-body movements in a setting without an explicit visual point of reference. This could be done by presenting the stimuli acoustically rather than visually. For example, headphones or an audio system with several speakers could be used so that no specific spatial dimension is perceived as more salient than the others.

Thirdly, our experimental setting did not allow for an option of no-movement, i.e., to remain in the starting position in the center of the pad after the presentation of the life-event. Therefore, although the study was framed within a free-choice paradigm, i.e., by choosing the spatial target and moving there, the lack of a no-movement option can also lead to seeing the paradigm as a force-choice task in the sense that participants were forced to move. Accordingly, it might be meaningful to examine whether, regardless of the emotion associated with the personal event, participants are willing to move at all. For example, it could be the case that, although a presented stimulus is negative, participants are not willing to avoid it. Moreover, this extension of the experimental setting, i.e., the possibility to remain in the middle of the dance pad, could be additionally combined with the presentation of neutral stimuli. For example, the study by Marmolejo-Ramos et al. (2017) showed that people tend to freely assign the word “surprise,” which may be seen as neutral, to central areas on a piece of paper. Therefore, it is plausible that neutral emotional events stimulate no-movement options, i.e., in our setting remaining on the center of the response pad. Accordingly, it could be expected that personal life events potentially perceived as neutral (e.g., shopping) will be more related to the no-movement option than positive and negative events.

Finally, our experimental setting only allowed to perform a single step. A possible extension of our paradigm could be to allow participants to freely walk in one of the directions. This could be done by positioning the eight spatial targets more distant from the initial neutral location and to enable to stop freely. In that way, beyond choosing a spatial target, it would be possible to track “walking distances,” namely the distance that participants cover regarding the presented stimuli. That would also enable the calculation of distances, for example by counting the performed steps for each event or using motion tracking devices. Besides tracking steps or movement trajectories, it could also be of interest to track other behavioral parameters such as the time associated with gait initiation, i.e., time needed to start the movement after the presentation of the life-event, as some studies investigating movement patterns associated with approach-avoidance behaviors do (e.g., Naugle et al., 2010). Moreover, this aspect could serve to potentially provide further insights within that research area as these studies do not typically account for several spatial directions simultaneously. For example, it could be hypothesized that participants would initiate movements faster on the more salient sagittal spatial dimension than on the other less salient spatial dimensions. Accordingly, it could also be examined if the diagonal spatial dimension is more salient as a result of connecting the sagittal with the lateral spatial dimension.

### Implications for Clinical Research

The present study did not directly address clinical applications. However, we believe that our experimental paradigm and related findings could serve to establish synergies between experimental and clinical research. In the Supplementary Appendix (see Supplementary Material), we will enumerate potential connections with some applied domains. We will discuss (a) how the type of paradigm used in our study could be beneficially included in intervention methods as the sensorimotor psychotherapy, (b) how bodily movements and particularly arm-movements, framed within approach-avoidance tasks, have been successfully used in programs aimed to modify psychological processes involved in addiction to unhealthy substances (alcohol or tobacco abuse), phobias (spider and social phobia) and autism, and (c) how to extend these approach-avoidance tasks so that the setting could involve not just arm movements but also the whole body. We will also discuss, first, why using a whole-body-movement paradigm could be a smother start in therapy, being potentially motivating for a broader type of patients and thus, leading to lesser therapy drop-outs and, second, how personalizing stimuli might contribute to enhancing the effectivity of approach-avoidance training (see Supplementary Appendix 1).

## Conclusion

A novel free-choice directional step paradigm is introduced in this study to explore whether an how the processing of affective concepts related to personal life events might resonate with whole-body actions in line with associations between emotional valence and lateral space (for right-handers: right-positive and left-negative) and approach-avoidance behaviors (forward-positive and backwards-negative). Since such associations are typically investigated independently one of each other and mostly relying on manual responses, here, right-handed participants were to freely step toward eight different directions (front, back, right, left, front–right, front–left, back–right, and back–left). Our findings indicate that approach-avoidance behaviors and space-valence associations across laterality are highly interwoven so that front–right and back–left are diagonal spatial dimensions strongly associated with the processing of positive and negative personal information, respectively. We argue that this type of experimental paradigm might serve to further connect experimental research within embodiment fields and clinical research addressing bodily experiences as a pivotal therapeutic component. Clinical implications are considered in more detail in the Supplementary Materials.

## Data Availability Statement

All datasets generated for this study are included in the Supplementary Files.

## Ethics Statement

Ethical review and approval was not required for the study on human participants in accordance with the local legislation and institutional requirements. The patients/participants provided their written informed consent to participate in this study.

## Author Contributions

SF and LK designed and planned the experiment. SF and JM were involved in planning and supervising the work. LK processed the experimental data. LK and SC-T designed the figures and tables and performed the statistical analysis. SF, LK, SC-T, and JM wrote the manuscript. All authors provided critical feedback and helped in every stage of the research, analysis, and manuscript.

## Conflict of Interest

The authors declare that the research was conducted in the absence of any commercial or financial relationships that could be construed as a potential conflict of interest.

## Footnotes

1 GEE account for correlated structures characteristic of repeated measurements such as the within-subjects factor valence category in the current study. Additionally, each participant responded to 12 personal events, hence, the trials were also nested within each participant. Moreover, in the regression model the estimates of seven spatial targets are compared against another spatial target used as reference. Here, front–right and back–left are used as reference categories with regard to positive and negative events, respectively.

## References

[B1] Ballinger G. A. (2004). Using generalized estimating equations for longitudinal data analysis. *Organ. Res. Methods* 7 127–150. 10.1177/1094428104263672

[B2] Barsalou L. W. (2008). Grounded cognition. *Ann. Rev. Psychol.* 59 617–645. 10.1146/annurev.psych.59.103006.093639 17705682

[B3] Borghi A. M. Binkofski F. Castelfranchi C. Cimatti F. Scorolli C. Tummolini L. (2017). The challenge of abstract concepts. *Psychol. Bull.* 143 263–292. 10.1037/bul0000089 28095000

[B4] Bouman D. Stins J. F. (2018). Back off! the effect of emotion on backward step initiation. *Hum. Mov. Sci.* 57 280–290. 10.1016/j.humov.2017.09.006 28919167

[B5] Brookshire G. Casasanto D. (2012). Motivation and motor control: hemispheric specialization for approach motivation reverses with handedness. *PLoS One* 7:e36036. 10.1371/journal.pone.0036036 22563436 PMC3338572

[B6] Brookshire G. Graver C. Casasanto D. (2013). Motor asymmetries predict neural organization of emotion. *Proc. Ann. Meet. Cogn. Sci. Soc.* 35 245–250.

[B7] Casasanto D. (2009). Embodiment of abstract concepts: good and bad in right-and left-handers. *J. Exp. Psychol. Gen.* 138 351–365. 10.1037/a0015854 19653795

[B8] Casasanto D. (2014). “Bodily relativity,” in *Handbook of Embodied Cognition*, ed. Shapiro L. (New York, NY: Routledge), 108–117.

[B9] Casasanto D. Henetz T. (2012). Handedness shapes children’s abstract concepts. *Cogn. Sci.* 36 359–372. 10.1111/j.1551-6709.2011.01199.x 21916951

[B10] Castaño E. Gilboy E. Feijóo S. Serrat E. Rostan C. Hilferty J. (2018). Hand position and response assignment modulate the activation of the valence−space conceptual metaphor. *Cogn. Sci.* 42 2342–2363. 10.1111/cogs.12669 30101555

[B11] Cervera-Torres S. Ruiz Fernández S. Lachmair M. Riekert M. Gerjets P. (2019). Altering emotions near the hand: approach-avoidance swipe interactions modulate the perceived valence of emotional pictures. *Emotion* 10.1037/emo0000651 [Epub ahead of print] . 31414834

[B12] Crawford L. E. (2009). Conceptual metaphors of affect. *Emot. Rev.* 1 129–139. 10.1177/1754073908100438

[B13] Cretenet J. Dru V. (2008). A neurobehavioral investigation into judgmental processes: effect of bilateral motor behaviors. *Brain Cogn.* 68 81–91. 10.1016/j.bandc.2008.03.002 18400351

[B14] D’Argembeau A. Van der Linden M. (2004). Phenomenal characteristics associated with projecting oneself back into the past and forward into the future: influence of valence and temporal distance. *Conscious. Cogn.* 13 844–858. 10.1016/j.concog.2004.07.007 15522635

[B15] Davis J. I. Gross J. J. Ochsner K. N. (2011). Psychological distance and emotional experience: what you see is what you get. *Emotion* 11 438–444. 10.1037/a0021783 21500912

[B16] de la Vega I. De Filippis M. Lachmair M. Dudschig C. Kaup B. (2012). Emotional valence and physical space: limits of interaction. *J. Exp. Psychol. Hum. Percept. Perform.* 38 375–385. 10.1037/a0024979 21928929

[B17] de la Vega I. Dudschig C. De Filippis M. Lachmair M. Kaup B. (2013). Keep your hands crossed: the valence-by-left/right interaction is related to hand, not side, in an incongruent hand–response key assignment. *Acta Psychol.* 142 273–277. 10.1016/j.actpsy.2012.12.011 23376138

[B18] de la Vega I. Graebe J. Härtner L. Dudschig C. Kaup B. (2015). Starting off on the right foot: strong right-footers respond faster with the right foot to positive words and with the left foot to negative words. *Front. Psychol.* 6:292. 10.3389/fpsyg.2015.00292 25852609 PMC4367177

[B19] Dudschig C. de la Vega I. Kaup B. (2015). What’s up? emotion-specific activation of vertical space during language processing. *Acta Psychol.* 156 143–155. 10.1016/j.actpsy.2014.09.015 25454886

[B20] Eder A. B. Hommel B. (2013). Anticipatory control of approach and avoidance: an ideomotor approach. *Emot. Rev.* 5 275–279. 10.1177/1754073913477505

[B21] Elliot A. J. (2006). The hierarchical model of approach-avoidance motivation. *Motivat. Emot.* 30 111–116. 10.1111/j.1467-6494.2006.00410.x 16958703

[B22] Fawver B. Hass C. J. Park K. D. Janelle C. M. (2014). Autobiographically recalled emotional states impact forward gait initiation as a function of motivational direction. *Emotion* 14 1125–1136. 10.1037/a0037597 25151514

[B23] Freddi S. Brouillet T. Cretenet J. Heurley L. P. Dru V. (2016). A continuous mapping between space and valence with left-and right-handers. *Psychon. Bull. Rev.* 23 865–870. 10.3758/s13423-015-0950-0 26428669

[B24] Freina L. Baroni G. Borghi A. M. Nicoletti R. (2009). Emotive concept nouns and motor responses: attraction or repulsion? *Mem. Cogn.* 37 493–499. 10.3758/MC.37.4.493 19460955

[B25] Friendly M. (1994). Mosaic displays for multi-way contingency tables. *J. Am. Stat. Assoc.* 89 190–200. 10.1080/01621459.1994.10476460

[B26] Fuchs T. Koch S. C. (2014). Embodied affectivity: on moving and being moved. *Front. Psychol.* 5:508. 10.3389/fpsyg.2014.00508 24936191 PMC4047516

[B27] Greiner B. (2004). “An online recruitment system for economic experiments,” in *Forschung Und Wissenschaftliches Rechnen 2003, GWDG Bericht 63*, eds Kremer K. Macho V. (Göttingen: Gesellschaft für wissenschaftliche Datenverarbeitung mbH), 79–93.

[B28] Guimaraes P. (2004). Understanding the multinomial-Poisson transformation. *Stata J.* 4 265–273. 10.1177/1536867x0400400304

[B29] Harmon-Jones E. Gable P. A. Peterson C. K. (2010). The role of asymmetric frontal cortical activity in emotion-related phenomena: a review and update. *Biol. Psychol.* 84 451–462. 10.1016/j.biopsycho.2009.08.010 19733618

[B30] Hartigan J. A. Kleiner B. (1984). A mosaic of television ratings. *Am. Stat.* 38 32–35. 10.1080/00031305.1984.10482869

[B31] Koch S. C. Glawe S. Holt D. V. (2011). Up and down, front and back. *Soc. Psychol.* 42 214–224.

[B32] Kong F. (2013). Space–valence associations depend on handedness: evidence from a bimanual output task. *Psychol. Res.* 77 773–779. 10.1007/s00426-012-0471-7 23288148

[B33] Lachmair M. Fernandez S. R. Bury N. A. Gerjets P. Fischer M. H. Bock O. L. (2016). How body orientation affects concepts of space, time and valence: functional relevance of integrating sensorimotor experiences during word processing. *PLoS One* 11:e0165795. 10.1371/journal.pone.0165795 27812155 PMC5094761

[B34] Macnamara A. Keage H. A. D. Loetscher T. (2018). Mapping of non-numerical domains on space: a systematic review and meta-analysis. *Exp. Brain Res.* 236 335–346. 10.1007/s00221-017-5154-6 29279982

[B35] Marmolejo-Ramos F. Correa J. C. Sakarkar G. Ngo G. Ruiz-Fernández S. Butcher N. (2017). Placing joy, surprise and sadness in space: a cross-linguistic study. *Psychol. Res.* 81 750–763. 10.1007/s00426-016-0787-9 27431389 PMC5486563

[B36] Marmolejo-Ramos F. Tirado C. Arshamian E. Vélez J. I. Arshamian A. (2018). The allocation of valenced concepts onto 3D space. *Cogn. Emot.* 32 709–718. 10.1080/02699931.2017.1344121 28657517

[B37] Miles L. K. Christian B. M. Masilamani N. Volpi L. Macrae C. N. (2014). Not so close encounters of the third kind: visual perspective and imagined social interaction. *Soc. Psychol. Personal. Sci.* 5 558–565. 10.1177/1948550613511500

[B38] Milhau A. Brouillet T. Brouillet D. (2012). Bidirectional influences of emotion and action in evaluation of emotionally-connoted words. *Biolinguistics* 6 417–432.

[B39] Milhau A. Brouillet T. Dru V. Coello Y. Brouillet D. (2015). Valence activates motor fluency simulation and biases perceptual judgment. *Psychol. Res.* 81 795–805. 10.1007/s00426-016-0788-8 27417215

[B40] Nair S. Sagar M. Sollers J. III Consedine N. Broadbent E. (2015). Do slumped and upright postures affect stress responses? A randomized trial. *Health Psychol.* 34 632–641. 10.1037/hea0000146 25222091

[B41] Naugle K. M. Joyner J. Hass C. J. Janelle C. M. (2010). Emotional influences on locomotor behavior. *J. Biomech.* 43 3099–3103. 10.1016/j.jbiomech.2010.08.008 20828698

[B42] Niedenthal P. M. (2007). Embodying emotion. *Science* 316 1002–1005. 10.1126/science.1136930 17510358

[B43] Niedenthal P. M. Winkielman P. Mondillon L. Vermeulen N. (2009). Embodiment of emotion concepts. *J. Personal. Soc. Psychol.* 96 1120–1136. 10.1037/a0015574 19469591

[B44] Oh S. Carriere K. C. Park T. (2008). Model diagnostic plots for repeated measures data using the generalized estimating equations approach. *Comput. Stat. Data Anal.* 53 222–232. 10.1016/j.csda.2008.07.022

[B45] Phaf R. H. Mohr S. E. Rotteveel M. Wicherts J. M. (2014). Approach, avoidance, and affect: a meta-analysis of approach-avoidance tendencies in manual reaction time tasks. *Front. Psychol.* 5:378. 10.3389/fpsyg.2014.00378 24847292 PMC4021119

[B46] Seibt B. Neumann R. Nussinson R. Strack F. (2008). Movement direction or change in distance? self-and object-related approach–avoidance motions. *J. Exp. Soc. Psychol.* 44 713–720. 10.1016/j.jesp.2007.04.013

[B47] Sharpe D. (2015). Your chi-square test is statistically significant: now what? *Pract. Assess., Res. Eval.* 20 1–10. 10.1186/ar3121 20738855 PMC2945065

[B48] Stins J. F. Beek P. J. (2011). Organization of voluntary stepping in response to emotion-inducing pictures. *Gait Posture* 34 164–168. 10.1016/j.gaitpost.2011.04.002 21549605

[B49] Van Boven L. Kane J. McGraw A. P. Dale J. (2010). Feeling close: emotional intensity reduces perceived psychological distance. *J. Personal. Soc. Psychol.* 98 872–885. 10.1037/a0019262 20515244

[B50] Vicario C. M. Rumiati R. I. (2014). Left-right compatibility in the processing of trading verbs. *Front. Behav. Neurosci.* 8:16. 10.3389/fnbeh.2014.00016 24478662 PMC3904129

