# Supplementary material for: Moved by Emotions: Affective Concepts Representing Personal Life Events Induce Freely Performed Steps in Line With Combined Sagittal and Lateral Space-Valence Associations
Source: Front Psychol. 2019 Dec 11;10:2787. doi: 10.3389/fpsyg.2019.02787 (PMC6917595; doi:10.3389/fpsyg.2019.02787)
Supplement: Supplementary file 2 [file Table_1.docx]

*Table S1*. Poisson regression estimates concerning the distribution of steps after exclusion of extreme observations. Estimates of valence category, spatial targets and their interactions are interpreted in relation to the Front-right spatial target and positive events.

|  |  | *B* | | *Std. Error* | | | *95% C.I. (B)* | | *Wald χ^2^  df Sig.* | | | | *Exp(B)* | | *1/ Exp(B)* | |
| --- | --- | --- | --- | --- | --- | --- | --- | --- | --- | --- | --- | --- | --- | --- | --- | --- |
|  | (Intercept) | | -2.386 | | .3874 | -3.146 | | -1.627 | 37.933 | 1 | .000 | .092 | | - | |  |
|  | Age | | -.003 | | .0036 | -.011 | | .004 | .915 | 1 | .339 | .997 | | - | |  |
|  | Gender | | -.043 | | .0415 | -.124 | | .038 | 1.068 | 1 | .301 | .958 | | - | |  |
|  | | Valence category | | 2.562 | | .4076 | 1.763 | | 3.361 | 39.525 | 1 | .000 | 12.965 | | - | |
|  | | Spatial target: | |  | |  |  | |  |  |  |  |  | |  | |
|  | | Front | | .893 | | .1666 | .567 | | 1.220 | 28.746 | 1 | .000 | 2.443 | | - | |
|  | | F-Left | | -.732 | | .1755 | -1.076 | | -.388 | 17.393 | 1 | .000 | .481 | | 2.08 | |
|  | | Right | | -1.051 | | .2785 | -1.597 | | -.505 | 14.233 | 1 | .000 | .350 | | 2.86 | |
|  | | Left | | -1.460 | | .2867 | -2.022 | | -.898 | 25.938 | 1 | .000 | .232 | | 4.31 | |
|  | | B-Right | | -2.979 | | .5029 | -3.964 | | -1.993 | 35.082 | 1 | .000 | .051 | | 19.61 | |
|  | | B-Left | | -2.571 | | .4062 | -3.367 | | -1.775 | 40.058 | 1 | .000 | .076 | | 13.16 | |
|  | | Back | | -1.234 | | .2589 | -1.741 | | -.727 | 22.723 | 1 | .000 | .291 | | 3.44 | |
|  | | Valence*Spatial Target: | |  | |  |  | |  |  |  |  |  | |  | |
|  | | Valence*Front | | -1.013 | | .4409 | -1.877 | | -.149 | 5.281 | 1 | .022 | .363 | | 2.75 | |
|  | | Valence*F-Left | | -1.564 | | .5405 | -2.623 | | -.504 | 8.371 | 1 | .004 | .209 | | 4.78 | |
|  | Valence*Right | | -2.854 | | .5234 | -3.880 | | -1.829 | 29.741 | 1 | .000 | .058 | | 17.24 | |  |
|  | Valence*Left | | -3.236 | | .4923 | -4.201 | | -2.271 | 43.215 | 1 | .000 | .039 | | 25.64 | |  |
|  | Valence*B-Right | | -4.860 | | .7788 | -6.387 | | -3.334 | 38.944 | 1 | .000 | .008 | | 125 | |  |
|  | Valence*B-Left | | -5.263 | | .6154 | -6.470 | | -4.057 | 73.142 | 1 | .000 | .005 | | 200 | |  |
|  | Valence*Back | | -4.339 | | .5138 | -5.346 | | -3.332 | 71.334 | 1 | .000 | .013 | | 76.92 | |  |

*Table S2*. Poisson regression estimates concerning the distribution of step after exclusion of extreme observations. Estimates of valence category, spatial targets and their interactions are interpreted in relation to the Back-left spatial target and negative events.

|  |  | *B* | | *Std. Error* | | | *95% C.I. (B)* | | *Wald χ^2^ df Sig.* | | | | *Exp(B)* | | *1/ Exp(B)* | |
| --- | --- | --- | --- | --- | --- | --- | --- | --- | --- | --- | --- | --- | --- | --- | --- | --- |
|  | (Intercept) | | -2.395 | | .3948 | -3.169 | | -1.621 | 36.789 | 1 | .000 | .091 | | - | |  |
|  | Age | | -.003 | | .0036 | -.011 | | .004 | .915 | 1 | .339 | .997 | | - | |  |
|  | Gender | | -.043 | | .0415 | -.124 | | .038 | 1.068 | 1 | .301 | .958 | | - | |  |
|  | | Valence category | | 2.701 | | .4180 | 1.882 | | 3.520 | 41.762 | 1 | .000 | 14.898 | | - | |
|  | | Spatial target: | |  | |  |  | |  |  |  |  |  | |  | |
|  | | Front | | -.786 | | .2308 | -1.239 | | -.334 | 11.606 | 1 | .001 | .456 | | 2.19 | |
|  | | F-Right | | -2.693 | | .4102 | -3.497 | | -1.889 | 43.081 | 1 | .000 | .068 | | 14.70 | |
|  | | F-Left | | -1.861 | | .3187 | -2.485 | | -1.236 | 34.093 | 1 | .000 | .156 | | 6.41 | |
|  | | Right | | -.889 | | .2374 | -1.354 | | -.424 | 14.022 | 1 | .000 | .411 | | 2.43 | |
|  | | Left | | -.916 | | .2242 | -1.356 | | -.477 | 16.709 | 1 | .000 | .400 | | 2.5 | |
|  | | B-Right | | -.811 | | .1800 | -1.164 | | -.458 | 20.292 | 1 | .000 | .444 | | 2.25 | |
|  | | Back | | .413 | | .1836 | .053 | | .773 | 5.056 | 1 | .025 | 1.511 | | - | |
|  | | Valence*Spatial Target: | |  | |  |  | |  |  |  |  |  | |  | |
|  | | Valence*Front | | -4.250 | | .4988 | -5.228 | | -3.273 | 72.606 | 1 | .000 | .014 | | 71.43 | |
|  | | Valence*F-Right | | -5.263 | | .6154 | -6.470 | | -4.057 | 73.142 | 1 | .000 | .005 | | 200 | |
|  | Valence*F-Left | | -3.700 | | .6413 | -4.957 | | -2.443 | 33.287 | 1 | .000 | .025 | | 40 | |  |
|  | Valence*Right | | -2.409 | | .5303 | -3.448 | | -1.370 | 20.640 | 1 | .000 | .090 | | 11.11 | |  |
|  | Valence*Left | | -2.027 | | .4888 | -2.985 | | -1.069 | 17.196 | 1 | .000 | .132 | | 7.57 | |  |
|  | Valence*B-Right | | -.403 | | .5376 | -1.457 | | .650 | .563 | 1 | .453 | .668 | | 1.49 | |  |
|  | Valence*Back | | -.924 | | .4567 | -1.819 | | -.029 | 4.094 | 1 | .043 | .397 | | 2.52 | |  |
