# Supplementary material for: Moved by Emotions: Affective Concepts Representing Personal Life Events Induce Freely Performed Steps in Line With Combined Sagittal and Lateral Space-Valence Associations
Source: Front Psychol. 2019 Dec 11;10:2787. doi: 10.3389/fpsyg.2019.02787 (PMC6917595; doi:10.3389/fpsyg.2019.02787)
Supplement: Supplementary file 3 [file Table_2.DOCX]

**Appendix 1**

**Implications for clinical research**

The present study did not directly address clinical applications. However, we believe that our experimental paradigm and related findings could serve to extend the synergies between experimental and clinical research. In the following, we will describe potential connections within some applied domains.

Potential synergies regarding therapy applications:

*Sensorimotor psychotherapy*

Sensorimotor psychotherapy is an intervention model that has been used for the treatment of people with post-traumatic stress disorder (PTSD; Ogden, Minton, & Pain, 2006). The main contribution of this model is the inclusion of somatic interventions at the same level as emotional or cognitive ones. By including the body as a primary way of intervention in the reprocessing of memories of trauma-events, therapists are able to directly work on bodily sensations and movements, potentially influencing patients’ symptoms by facilitating changes in cognitions, emotions, beliefs and relational capabilities of the patients. Specifically, the sensorimotor psychotherapy is structured in three main phases. The first phase is aimed at identifying somatic reactions linked to hyper-activation or hypo-activation. During the second phase, the traumatic events are recalled. The goals of this phase are, on the one hand, to reduce the patients’ avoidance and phobic reactions toward traumatic memory-events and, on the other hand, to explore and implement tendencies of action that help to regulate physiological responses produced by inadequate defensive reactions associated with those events. The third phase is aimed at normalizing the daily functioning of the patient in those areas that could have been affected during the disorder, using the strategies and knowledge developed during the two previous phases. Based on this reasoning, it is possible that this type of interventions could benefit from the type of paradigm used in our study. For example, once the traumatic events are detected and recalled, it could be possible to represent them by means of words or short sentences, thus making them more visually (or acoustically) explicit. Then, in line with our study, the response pad could be used to explore types of directional movements initially associated with the traumatic events. Finally, it could be interesting to examine the potential for specific directional movements to reduce physiological symptoms (e.g., anxiety levels).

Potential synergies regarding clinical disorders:

*Substance abuse disorder: Alcohol and tobacco abuse*

Bodily movements and particularly arm-movements, framed within approach-avoidance tasks, have been successfully used in programs aimed to modify psychological processes involved in addiction to unhealthy substances such as alcohol or tobacco.

a) Alcohol abuse: A study by Wiers et al. (2011) used approach-avoidance arm movements within a module of Cognitive Bias Modification (CBM; MacLeod et al., 2002). The goal of the CBM in this context was to modify approach tendencies towards alcohol among people suffering from alcoholism. In this study, patients were randomly assigned either to an experimental or a control condition. Patients in the experimental condition were trained to consistently push a joystick in response to alcoholic drink pictures and pull a joystick in response to pictures of non-alcoholic drinks, whereas patients in the control conditions were either not trained to avoid such pictures at all or received sham training. Only the patients in the experimental condition showed a modified bias for alcohol consumption, changing from a small approach bias to a strong avoidance bias for alcoholic drinks. Additionally, when those participants were evaluated one year later, the authors found that relapse rates among the experimental group were lower (46%) than among the control group (59%). A further study by Eberl et al. (2013) replicated these findings and extended them, by showing that older patients and patients with a strong approach bias profited most from the CBM (see also Wiers et al., 2010 for a study examining approach-avoidance training with hazardous drinkers).

b) Tobacco abuse: A further study by Wittekind et al. (2015) tested the approach-avoidance training with smokers. In their study, participants had to react to smoking-related or neutral pictures by respectively pushing or pulling the computer-mouse. Results showed that participants reduced their cigarette consumption after this training, thus suggesting, that this setting can be applied as an intervention. A similar result was found for smokers undergoing inpatient psychiatric treatment (Machulska et al., 2016). The results showed reduced smoking behaviour for the pushing condition. Additionally, a three-month follow up showed that the training led to a larger reduction of cigarette consumption.

It should be noted that in approach-avoidance settings, as described above, forward movements (i.e., push movement) were mostly interpreted as an avoidance gesture in order to push away the presented stimuli from the own body (i.e., self-as-reference). Transferring these designs into a whole-body movement setting, which includes moving towards or away from the emotional-laden stimuli, as in this case pictures that are related or unrelated to alcoholic drinks and smoking, could be an approach to examine whether this would lead to a stronger avoidance bias for alcohol. Additionally, the setting could turn to be more realistic if patients move from or away real drinks or cigarettes, as it is the case in every-day situations, for example when they have to go to the supermarket. Although these types of interventions are mostly based on visual stimuli it seems reasonable to use a whole-body movement paradigm such as the one in our study to investigate, for example, differences in alcohol or tobacco avoidance tendencies as a function of movement direction and particularly the diagonal back-left direction for right-handed participants.

*Anxiety disorder: Spider phobia and social phobia*

Since, one of the factors in the maintenance of phobia is the avoidance of the aversive stimuli and in therapy, one of the most useful interventions is to expose patients to the fear-inducing stimuli (i.e., exposure treatment/therapy), the question arises whether it is possible to reframe or at least lessen the negativity of fear-inducing (or aversive) stimuli by performing whole-body approach movements towards them. In standard phobia treatments, patients have to remain in the fearful situation until the fear decreases significantly. If the fear is very high, the appropriate therapy could start with imagination tasks including fear-inducing stimuli (e.g., to imagine a spider nearby the patient); the second stage would, then, include the real exposure of the fear-inducing stimuli (i.e., systematic desensitization). Thus, it would be interesting to explore whether the setting of the current study could be used as an approach-avoidance training in addition to the standard therapy of phobia (for a critical study concerning approach-avoidance training with fear-conditioned stimuli see Mertens et al., 2018).

a) Spider phobia: For example, patients suffering from spider phobia could approach the word “spider” or spider pictures in a first stage and later, in a second stage, a real spider placed in a terrarium, i.e., in this case trying to lessen the phobia of spiders by linking the embodied positive connotation of approaching the fear-inducing stimulus (see also stages of an exposure therapy; Rowe & Craske, 1998). A recent study provided evidence that fearful individuals who approached spider pictures first were less anxious in a second stage characterized by encountering real spiders compared to individuals that did not approach spider pictures (Jones et al., 2013). Specifically, experimental conditions where individuals with high spider fear performed approach movements toward spider pictures by using a joystick subsequently manifested lower anxiety levels after approaching real tarantulas in a more realistic scenario, i.e., by moving towards the spiders. Although this study shows that approaching a fear-induced stimulus with arm-movement using a joystick lead to a decrease of anxiety levels when confronted with the real stimulus it can be expected that performing whole-body movements instead of arm-movements in a first stage could lead to a higher decrease in fear when confronted with the real fear-inducing stimulus.

b) Social phobia: This setting could also be of interest in the training of patients suffering from social phobia. In this case, patients could be confronted with pictures of faces or facial expressions, or also with pictures including explicit fear-inducing situations typical for socially anxious individuals (e.g., speech in front of an audience). In the same rationale, training to approach the fear-inducing stimuli by whole-body movements could lead to reduced fear when individuals are confronted with the real fear-inducing situation or in the best case lessen the avoidance of real fear-inducing situations. Previous research revealed that socially anxious people show stronger avoidance tendencies for smiling than non-anxious people but at the same time did not differ in the valence ratings of emotional facial expression from non-anxious people, thus, evaluating smiling faces as positive as non-anxious people did (e.g., Heuer et al., 2007). Accordingly, the approach-avoidance setting can be especially effective when training patients suffering from social phobia because these patients positively evaluate the fear-induced stimuli in contrast to patients, suffering from other phobias (i.e., spider phobia patients that show negativity towards the fear-inducing stimuli that they had to approach). Accordingly, it would be hypothesized that approach training could provide a stronger decrease in social anxiety when facing the situation. In a study by Rinck et al. (2013) individuals with high social anxiety were trained either to approach or avoid pictures of smiling faces by pushing or pulling a joystick that led to shrinking or growing of the presented pictures, respectively. Results showed that participants, trained to approach smiling faces, approached females (but not males) faces faster than participants, trained to avoid smiling faces. In addition, subsequent to their training, participants were in a better mood after being asked to give a videotaped self-presentation and showed lower anxiety after the self-presentation that they had to perform after the training (see also Taylor & Amir, 2012 for an approach training using smiling and neutral faces). It is important to note that studies examining the influence of approach-based training on phobias mostly used arm-movements and not whole-body movements. As seen in the present study, participants positioned themselves more often in the diagonal front position as in the straight front position when a positive life-event was presented. Thus, it would be interesting to examine whether fear could be reduced faster when patients move to the front-right position instead of the straight front position.

*Autistic spectrum disorder*

There is a general agreement that people suffering from autism are characterized, among other features, by having problems to recognize social cues such as emotional facial expressions and bodily postures (e.g., Reed et al., 2007). This could explain why this group of people has difficulties to naturally produce meaningful gestures and bodily expressions, which tend to be rather mechanical (cf. Donellan et al., 2013). Consequently, it is reasonable that the role of bodily movements is increasingly addressed in therapies directed to autistic populations. For example, the findings by Koch et al. (2015) suggest that young autistic adults may benefit from whole-body mirroring movements so that they report improved body awareness, self-other awareness, and psychological well-being. Studies based on kinematic bodily parameters, such as movement velocity, are also being explored for their potential to detect emotional features in autistic children via gait-pattern analyses (e.g., Jamil et al., 2015). Moreover, with the rise of novel interactive technologies, the use of avatars has also been recently examined as a method for improving body mimicry (e.g., Forbes et al., 2016). Of course, the literature in this domain is much more extensive. Yet, based on the afore-mentioned studies, it would be feasible to adapt the experimental paradigm, used in the current study, to combine, for example within a gaming framework, the movement mimicry component with the gait-analyses. Avatars and, particularly, personalized ones such as avatars representing caretakers, relatives, or a self-avatar, could be used as the focus of reference to mimic movements. Although these are just loose ideas, we consider the potential that a paradigm based on “space-valence” gait movements might contribute to the field.

**Therapy setting**

From another perspective, using a whole-body-movement paradigm could be also a smother start in therapy than starting directly with exposure. Specifically, it is likely, that individuals, suffering e.g., from phobia, will consent faster to an approach-training than an exposure therapy so that it can be used as a first step and an ice breaker to initiate the therapy (e.g., Choy et al., 2007). Additionally, for patients undergoing psychiatric treatment, sometimes showing higher cognitive and somatic impairments, a setting as the one presented in our study can be easily performed by patients. It is also possible that a whole-body movement setting that can be set up in every room will motivate patients more than performing arm-movements in a computer-based environment. Especially for patients with greater cognitive impairments and for older patients, the whole-body movement setting can be more feasible and motivating than sitting in front of a computer and, thus, leading to lesser therapy drop-outs.

Furthermore, personalizing stimuli could contribute to enhancing the effectivity of the approach-avoidance training. Most studies, examining the influence of training, employed pictures of, for example, fear-inducing or addiction-related stimuli. Although research showed that pictures can induce the intentioned emotion recent research also showed that autobiographical stimuli have a higher impact (Fawver et al., 2014) because the stimuli are linked with memories and experiences that resonate with the body. For example, it seems possible that the training would lead to other outcomes if patients, suffering from alcoholism, were presented pictures of a more realistic scenario, i.e., bottles of the preferred drink compared to pictures or bottles of alcohol that they didn’t prefer. If one thinks in terms of diagnostics the use of personalized stimuli could also lead to more fine-grained information about, for example, the anxiety or the addiction of patients. Thus, implementing such personalized whole-body approach-avoidance training as a co-therapy could be of high value.

**References**

Choy, Y., Fyer, A. J., & Lipsitz, J. D. (2007). Treatment of specific phobia in adults. *Clinical Psychology Review, 27*(3), 266–286.

Donnellan, A. M., Hill, D. A., & Leary, M. R. (2013). Rethinking autism: implications of sensory and movement differences for understanding and support. *Frontiers in Integrative Neuroscience*, *6*:124.

Eberl, C., Wiers, R. W., Pawelczack, S., Rinck, M., Becker, E. S., & Lindenmeyer, J. (2013). Approach bias modification in alcohol dependence: do clinical effects replicate and for whom does it work best? *Developmental Cognitive Neuroscience, 4*, 38–51. doi: 10.1111/acer.12281

Fawver, B., Hass, C. J., Park, K. D., & Janelle, C. M. (2014). Autobiographically recalled emotional states impact forward gait initiation as a function of motivational direction. *Emotion*, 14(6), 1125–1136. doi: 10.1037/a0037597

Forbes, P. A., Pan, X., & Hamilton, A. F. D. C. (2016). Reduced mimicry to virtual reality avatars in autism spectrum disorder. *Journal of Autism and Developmental Disorders*, *46*(12), 3788-3797.

Heuer, K., Rinck, M., & Becker, E. S. (2007). Avoidance of emotional facial expressions in social anxiety: The approach–avoidance task. *Behaviour Research and Therapy*, *45*(12), 2990-3001.

Jamil, N., Khir, N. H. M., Ismail, M., & Razak, F. H. A. (2015). Gait-based emotion detection of children with autism spectrum disorders: a preliminary investigation. *Procedia Computer Science*, *76*, 342-348.

Jones, C. R., Vilensky, M. R., Vasey, M. W., & Fazio, R. H. (2013). Approach behavior can mitigate predominately univalent negative attitudes: Evidence regarding insects and spiders. *Emotion*, *13*(5), 989-996. doi: 10.1037/a0033164

Koch, S. C., Mehl, L., Sobanski, E., Sieber, M., & Fuchs, T. (2015). Fixing the mirrors: A feasibility study of the effects of dance movement therapy on young adults with autism spectrum disorder. *Autism*, *19*(3), 338-350.

Machulska, A., Zlomuzica, A., Rinck, M., Assion, H. J., & Margraf, J. (2016). Approach bias modification in inpatient psychiatric smokers*. Journal of Psychiatric Research, 76*, 44–51. doi: 10.1016/j.jpsychires.2015.11.015

MacLeod, C., Rutherford, E., Campbell, L., Ebsworthy, G., & Holker, L. (2002). Selective attention and emotional vulnerability: assessing the causal basis of their association through the experimental manipulation of attentional bias. *Journal of Abnormal Psychology, 111*(1), 107-123. doi: 10.1037/0021-843X.111.1.107

Mertens, G., Van Dessel, P., & De Houwer, J. (2018). The contextual malleability of approach-avoidance training effects: approaching or avoiding fear conditioned stimuli modulates effects of approach-avoidance training. *Cognition and Emotion, 32*(2), 341–349. doi: 10.1080/02699931.2017.1308315

Ogden, P., Minton, K., & Pain, C. (2006). *Trauma and the Body: A sensorimotor Approach to Psychotherapy.* New York, NY, US: WW Norton & Company.

Reed, C. L., Beall, P. M., Stone, V. E., Kopelioff, L., Pulham, D. J., & Hepburn, S. L. (2007). Brief report: Perception of body posture—what individuals with autism spectrum disorder might be missing. *Journal of Autism and Developmental Disorders*, *37*(8), 1576-1584.

Rinck, M., Telli, S., Kampmann, I., Woud, M. L., Kerstholt, M., te Velthuis, S., ... & Becker, E. S. (2013). Training approach-avoidance of smiling faces affects emotional vulnerability in socially anxious individuals. *Frontiers in Human Neuroscience, 7*:481. doi: 10.3389/fnhum.2013.00481

Rowe, M. K., & Craske, M. G. (1998). Effects of varied-stimulus exposure training on fear reduction and return of fear. *Behaviour Research and Therapy, 36*(7-8), 719–734. doi: 10.1016/S0005-7967(97)10017-1

Taylor, C. T., & Amir, N. (2012). Modifying automatic approach action tendencies in individuals with elevated social anxiety symptoms. *Behaviour Research and Therapy*, *50*(9), 529–536. doi: 10.1016/j.brat.2012.05.004

Wiers, R. W., Eberl, C., Rinck, M., Becker, E. S., & Lindenmeyer, J. (2011). Retraining automatic action tendencies changes alcoholic patients’ approach bias for alcohol and improves treatment outcome. *Psychological Science*, *22*(4), 490–497. doi: 0.1177/0956797611400615

Wiers, R. W., Rinck, M., Kordts, R., Houben, K., & Strack, F. (2010). Retraining automatic action‐tendencies to approach alcohol in hazardous drinkers. *Addiction, 105*(2), 279–287. doi: 10.1111/j.1360-0443.2009.02775.x

Wittekind, C. E., Feist, A., Schneider, B. C., Moritz, S., & Fritzsche, A. (2015). The approach-avoidance task as an online intervention in cigarette smoking: A pilot study. *Journal of Behavior Therapy and Experimental Psychiatry, 46*, 115–120. doi: 10.1016/j.jbtep.2014.08.006
